# Intracalvariosseous administration of donepezil microspheres protects against cognitive impairment by virtue of long-lasting brain exposure in mice

**DOI:** 10.7150/thno.100986

**Published:** 2024-10-14

**Authors:** Ji Hee Kang, Jin-Kyoung Yang, Kyo Hee Cho, O Hyun Lee, Hayoon Kwon, Sun Yeou Kim, Sehoon Kim, Young Tag Ko

**Affiliations:** 1 College of Pharmacy and Gachon Institute of Pharmaceutical Sciences, Gachon University, Incheon, 21936, Republic of Korea.; 2 Department of Chemical Engineering, Dong-Eui University, Busan, 47340, Republic of Korea.; 3 Chemical & Biological Integrative Research Center, Korea Institute of Science and Technology, Seoul, 02792, Republic of Korea.; 4 KU-KIST Graduate School of Converging Science and Technology, Korea University, Seoul, 02841, Republic of Korea.

**Keywords:** intraclavariosseous administration, brain drug delivery, BBB-bypassing route, Alzheimer's disease, Long-acting injectable PLGA microspheres

## Abstract

**Rationale:** Recent studies have demonstrated the direct connections between the skull bone marrow, meninges, and brain. In an effort to explore these connections for the purpose of brain drug delivery, we previously proposed the direct application of CNS drugs into the diploic space between the outer and inner cortex of the skull, namely, intracalvariosseous administration (ICO). It was successfully demonstrated that small molecular to large colloidal drugs can readily reach the brain after ICO in mice and rabbits. Here, we report that a single ICO of donepezil microspheres protects cognitive impairment in Alzheimer mouse models over a month-long period.

**Methods:** Donepezil-loaded long-acting microspheres (DPZ@LAM) were prepared with biodegradable poly(DL-lactide-co-glycolide) (PLGA). Pharmacokinetic study and behavioral test were performed to determine the brain exposure and therapeutic effects after ICO of DPZ@LAM in scopolamine-induced memory-deficient mice.

**Results:** DPZ@LAM were capable of a month-long and precisely controlled drug release. After a single ICO of DPZ@LAM, DPZ concentration in brain sustained above the effective therapeutic levels for four weeks. The long-lasting brain exposure also led to significantly recovered cognitive function in scopolamine-induced memory-deficient mice, along with decreased acetylcholinesterase activity and increased brain-derived neurotrophic factor.

**Conclusions:** ICO allows for BBB-bypassing brain drug delivery through the direct connection between the skull bone marrow and brain, providing an alternative approach for the treatment of neurodegenerative diseases with otherwise BBB impermeable CNS drugs.

## Introduction

Alzheimer's disease (AD), the most common neurodegenerative disorder, is characterized by cholinergic deficit and abnormal protein deposition in the central nervous system (CNS), inducing loss of cognitive function 1-3. In the worldwide population, AD accounts for 60-70% of all 55 million dementia cases 4, 5. Albeit numerous trials, only a few were clinically approved as AD therapeutics, including long-standing acetylcholinesterase (AchE) inhibitors (donepezil, rivastigmine, galantamine), glutamate regulators (memantine), and newly added anti Aβ monoclonal antibodies. Until marketing of monoclonal antibodies (Aducanumab, Lecanemab) in 2021 and 2023, the AchE inhibitors, indirect cholinergic agonists, have been sole therapeutics for AD treatment 6-8. Justified by the fact that the loss of the cholinergic neurons is a key pathologic feature in AD patient's cerebral cortex, AchE inhibitors have been widely accepted as standard pharmacotherapeutics for decades 9. Donepezil (DPZ) has been the most commonly prescribed AchE inhibitor for it is the only AchE inhibitor for all stages AD of mild to severe, whereas others are for mild to moderate stages 10. However, DPZ undergoes severe first-pass metabolism and exhibits low brain bioavailability with the average CSF:plasma percent ratio of 15.7% 11. This necessitates repeated administration of high doses (5 - 23 mg/day) to achieve and sustain therapeutic level in brains 11-13. As a result, patients experience severe adverse effects such as bradycardia, diarrhea, vomiting and insomnia, causing poor patient compliance 14, 15. Therefore, the strategy to increase brain exposure (brain bioavailability) and reduce the doses (intervals) minimizes adverse effects and improves patient compliance in AD treatment with DPZ.

As poor patient compliance being a major issue in AD treatment, long-acting microspheres (LAM) would provide a potentially effective solution 8, 16. LAM are capable of controlled and sustained release for longer period. This capacity allows LAM to maintain a steady-state concentration within therapeutic level, with fewer peak and trough fluctuation, thereby reducing drug adverse effects 17, 18. Biodegradable polymeric microspheres have been one of the most common types in LAM formulation due to the capability of customizing drug release at a controlled rate for a targeted period by regulating the polymer degradation 17, 19. Among the biodegradable polymers for LAM, poly(DL-lactide-co-glycolide) (PLGA) is a popular choice as a rate-controlling agent due to the abundant molecular pool of commercial availability and degraded to glycolic acid and lactic acid monomers. PLGA enable an extensive fine-tuning of drug release by various combinations of physicochemical properties such as molecular weight, lactide/glycolide ratio, and terminal groups 20-22.

Recent studies have reported the direct channels between the skull bone marrow and the meninges that allow the trafficking of immune cells and direct access of cerebrospinal fluid (CSF) to bone marrow niches 23-32. In an effort to explore these direct channels for brain drug delivery, we previously established intracalvariosseous administration (ICO), the direct drug application into the diploic space between the outer and inner cortex of the skull bone 33. The previous results demonstrated that ICO could enhance brain exposure level of small molecular CNS drugs several tens to a hundred times compared to IV injection in a day-long study with mice. In a month-long study with rabbits and infusion pump implants (under review), we also demonstrated that ICO readily delivers various molecules, including small molecular compounds, macromolecular nucleic acids and large colloidal nanoparticles to the brain. Together, we can conclude that the inner cortex of the skull is permeable to various sized molecules and ICO serves as a new blood-brain barrier (BBB)-bypassing route for brain drug delivery, particularly for drugs facing challenges in crossing the BBB 33.

**Figure 1:**
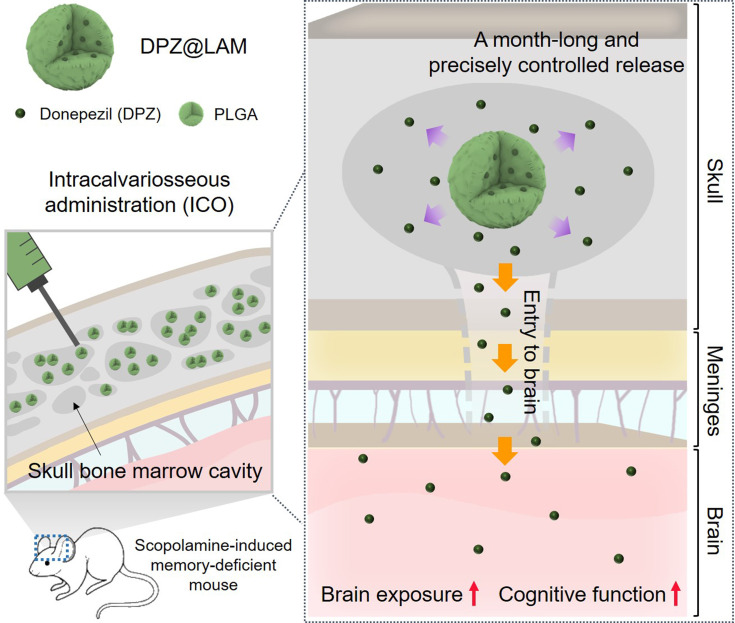
Schematic illustration of protecting against cognitive impairment after ICO of donepezil-loaded long-acting PLGA microspheres (DPZ@LAM) by long-lasting brain exposure in scopolamine-induced memory-deficient mouse models.

Here, we report that intracalvariosseous administration of donepezil-loaded long-acting microspheres protects against cognitive impairment through long-lasting brain exposure in mice (**Figure 1**). To advance one-step further toward the clinical translation, we aimed to apply the well-established ICO to donepezil with a better compliant dosage form. The pump implant in the previous study was replaced with long-acting injectable dosage form with no need for surgical procedure. Donepezil was loaded into long-acting PLGA microspheres capable of sustained and controlled release of a month-long duration and nano flow rate. Physicochemical properties of donepezil-loaded long-acting PLGA microspheres (DPZ@LAM) were characterized in terms of size distribution, morphology, encapsulation efficiency, internal structure, drug release, and microsphere erosion. After ICO of DPZ@LAM in mice, DPZ level in plasma, brain interstitial fluid (ISF), and whole brain were determined and compared to those after ICO of free DPZ. Furthermore, the efficacy of ICO of DPZ@LAM on cognitive performance was evaluated in terms of behavioral tests, immunoassay, and histological analysis in scopolamine-induced cognitive impairment mouse models.

## Materials and methods

### Materials

PLGA (7-17 kDa, lactide/glycolide ratio = 50/50), polyvinyl alcohol (PVA, 9-10 kDa, 80% hydrolyzed), and fluorescein isothiocyanate (FITC) were purchased from Sigma-Aldrich (St. Louis, MO, USA). Donepezil hydrochloride (DPZ) was obtained from TCI Chemicals (Tokyo, Japan). Ethyl acetate (EA) was bought from Duksan Pure chemical (Ansan, Korea). DiD was purchased from ThermoFisher scientific (Waltham, MA, USA). Deionized (DI) water was used for all experiments.

### Preparation of DPZ@LAM

DPZ@LAM was prepared by a double emulsion technique. Briefly, PLGA was dissolved in EA (4 w/v%). Aqueous 0.5% PVA solution containing DPZ was added to the PLGA solution at an aqueous/organic solvent volume ratio of 1:4. The mixture was sonicated using a probe-type sonicator (Scientz-IID, Scientz, Ningbo, China) with a pulse of 1 s-on/1 s-off at a power of 285 W. After sonication for 1 min, water-in-oil (W/O) emulsion was formed. An aqueous 0.5% PVA solution was then added to W/O emulsion and homogenized to prepared water-in-oil-in-water (W/O/W) emulsion. The resulting emulsion was agitated for 3 h at room temperature to evaporate EA. Prepared DPZ@LAM was washed five times with 0.5% (w/v) PVA/DI water solution and subsequently freeze-dried for the storage.

### Characterization of DPZ@LAM

The size and morphology of DPZ@LAM were analyzed by optical microscope (Nuance, PerkinElmer, USA) and scanning electron microscope (SEM, Regulus 8230, Hitachi, Tokyo, Japan). Size distribution of DPZ@LAM was obtained by microsize-particle analyzer (Mastersizer 3000, Malvern Panalytical Ltd., UK). UV/Vis absorbance spectra were collected with a UV/Vis spectrophotometer (Agilent 8453 UV-Visible spectroscopy system, Agilent Technologies, USA). Fluorescence microscopy was performed using a confocal microscope (Zeiss LSM 800, Carl Zeiss Microimaging Inc., NY, USA). Degradation of DPZ@LAM in 1X PBS (pH 7.4) depending on the incubation time was observed by SEM.

### DPZ encapsulation efficiency and loading capacity

The supernatant was collected in the purification process after double-emulsion preparation of DPZ@LAM. The absorbance of DPZ in the supernatant was measured using UV/Vis spectrometer, and then the concentration of DPZ was estimated on the basis of the UV absorption intensity using calibration curve established from standard solutions of DPZ in DI water. With this concentration, encapsulation efficiency (EE%) was calculated using the following an equation:

EE% = 
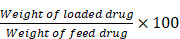


Loading capacity (LC%) was determined by dissolving DPZ@LAM in EA solution. The solution was sonicated for 2 h to ensure complete dissolution of DPZ@LAM. Next, the dissolved solution was filtrated, followed by measurement of UV/Vis absorption. The amount of DPZ was estimated with calibration curve derived from the standard solutions of DPZ in EA. The LC% was calculated using the following an equation:

LC% = 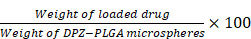


### *In vitro* cumulative drug release

The *in vitro* release study of DPZ@LAM was performed by using GeBa Flex-tube dialysis kit (8 kDa MWCO, Dialysis Membrane, Israel). DPZ@LAM of 1 mg was packed in a dialysis membrane tube and then the dialysis tube was individually immersed in 3 mL of 1X PBS (pH 7.4). The temperature was maintained at 37 ± 1 ºC and the receptor medium was perpetually stirred at 50 rpm to maintain the sink condition. At regular time intervals, the released samples (3 mL) were individually collected and replaced with a fresh buffer of same volume. The amount of DPZ release through membrane was quantified using LC-MS/MS.

### *In vivo* assessment of microdialysis probe recovery

A total of 6 kDa CMA 7 microdialysis probes (Havard apparatus, Holliston, MA, USA) were connected with PE/PVC tubing (0.6 ⅹ 1.6 mm) to a PHD ULTRA^TM^ syringe pump (Havard apparatus, Holliston, MA, USA). For equilibration of microdialysis probe, the inlet of the microdialysis probe was perfused with filtered artificial cerebrospinal fluid (aCSF) buffer containing 122 mM NaCl, 1.3 mM CaCl_2_, 1.2 mM MgCl_2_, 3.0 mM KH_2_PO_4_, and 25.0 mM NaHCO_3_ at a flow rate of 0.5 μL/min, and the outlet was kept in empty Eptube for 1 h, with the membrane of the microdialysis probe soaking in ethanol. To estimate *in vivo* recovery, retrodialysis was performed with aCSF containing DPZ 34. The *in vivo* recovery was calculated as equivalent to the loss upon retrodialysis using the following equation:

*In vivo* recovery 
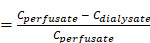


where C_perfusate_ is the compound concentration in the perfusate (inlet of the microdialysis probe), and C_dialysate_ is the compound concentration in the dialysate (outlet of the microdialysis probe). Recovery was utilized to calculate the C_ISF_, which is the concentration of the compound in the brain ISF, from the measured brain ISF dialysate concentration 35.

### Animals

All animal procedures were approved by the Institutional Animal Care and Use Committee at Gachon University, Republic of Korea and complied with the guideline for users of Animal Ethics Committee of Gachon University [Approval number: GUI-2022-IA0057-00]. The experiments were conducted with male BALB/c mice aged 5 weeks with a weight range of 20-25 g.

### ICO device installation in mice

We carried out skull thinning process in mice under anesthesia to simulate intracalvariosseous administration (ICO) as described in a previous study 33. Briefly, the right parietal bone of the skull (2.0 mm anterior/posterior (AP) and 2.0 mm medial/lateral (ML) from the bregma) was thinned to a thickness of 200 μm by a drill bit of 1.2 mm diameter using Robot stereotaxic (NeuroStar, Tubingen, Germany). After the skull thinning process, clearing skull bone dust, embedding ICO device in the thinned area, and suturing the skin were performed. After ICO device installation, 50 μL of saline was placed into the ICO device until the time of drug administration.

### *In vivo* brain exposure in mice

#### Implantation of guide cannula for microdialysis probe

Implantation of guide cannula for microdialysis probe in mice under anesthesia was performed as previously described 33. Briefly, a guide cannula was stereotactically implanted in the left hippocampus (2.0 mm anterior/posterior (AP), -1.5 mm medial/lateral (ML), and -2.0 mm dorsal/ventral from the bregma) through 1.2 mm-diameter hole in the skull. The guide cannula was secured with dental cement and the skin was sealed with sutures. The mice were then returned to their cages and allowed to recover for 1 day.

#### Drug administration

DPZ@LAM or free DPZ was applied in the pre-installed ICO device at a dose of 5 mg/kg as DPZ by replacing the pre-filled saline with the same volume of DPZ@LAM or free DPZ in 0.9% saline. DPZ@LAM solution was prepared at the same DPZ concentration as the free DPZ solution, based on the calculated LC value. The same dose of free DPZ was also administered by PO for control.

#### Sampling of brain microdialysates, plasma and whole brain

Bioanalytes sampling was performed as previously described 33. Briefly, after stabilization of microdialysis probes in ethanol with aCSF flow for 1 h, a probe was inserted into the guide cannula in mouse and then perfusates from the microdialysis probes were collected every 30 min after administration. Blood samples of 30 μL were collected from the saphenous vein at predetermined time after administration into 0.6 mL heparinized tubes. The samples were immediately centrifuged at 10,000 rpm for 10 min at 4°C to separate high-purity plasma from blood cells with discarding platelets, particulates and/or lipids 36-38. The whole brain was excised at final day post-administration and homogenized using Bioprep-24homogenizer (Bioand, Seoul, Korea) after adding PBS equivalent to the weight of each brain sample. Microdialysates, plasma and homogenized brain tissue samples were stored at -80°C until LC-MS/MS analysis.

#### Sampling of skull, dura, cortex, hippocampus and subcortex

The skull was carefully cut in the longitudinal plane, from the back of the head to the nose, ensuring no damage to the brain. The brain was gently extracted from the skull using forceps without scraping the inside of the skull. The brain was cut along the midline of the cerebrum (frontally) and the midline of the brainstem (posteriorly) using a razor. The cortex, hippocampus and subcortex were then isolated from left and right hemispheres using forceps and curved tweezers 39. The isolated tissues were homogenized using Bioprep-24homogenizer after adding PBS equivalent to the weight of each tissue sample. The dural meninges inside the skull are carefully isolated from the inner surface of the skull by edge-scratching with forceps 40. The skull was then cleaned by removing the skin, muscles, and non-relevant regions, leaving only the frontal, parietal, and interparietal bones. Finally, the skull was divided into left and right halves by cutting along the sagittal and frontal sutures with scissors. All samples were stored at -80°C until LC-MS/MS analysis.

#### LC-MS/MS analysis

DPZ was extracted from microdialysates, plasma, whole/dissected brain homogenates, dura and skull by liquid-liquid extraction (LLE) using ethyl acetate. Microdialysate samples (10 μL), plasma samples (10 μL) and brain homogenate samples (50 μL) were mixed with internal standard of 10 μL and ethyl acetate of 250 μL by vortexing for 2 min. The dura and skull samples were similarly mixed with internal standard of 10 μL and ethyl acetate of 250 μL, follwed by vortexing for 2 min and sonicating for 20 min. Following a 10-minute centrifugation at 10,000 rpm and 4°C, the organic layer was separated and dried under vacuum. Lastly, the dried residue was reconstituted in mobile phase of 20 μL (acetonitrile and 0.1% formic acid-water, 60:40%, v/v) and immediately analyzed by LC-MS/MS. An Agilent 6490 triple quadrupole mass spectrometer (MS) (Agilent Technologies, CA, USA) with an electrospray ionization (ESI) source was equipped with an Agilent LC 1100 series. An analytical Sepax BR-C18 (5 μm, 1.0 × 100 mm) column was used at 45°C under isocratic conditions of mobile phase (acetonitrile and 0.1% formic acid-water, 60:40%, v/v) at a flow rate of 0.10 mL/min. The analytes were identified by monitoring the precursor-to-product ion transitions of 380.1-91.1 for DPZ with collision energy of 36, using the positive multiple reaction monitoring (MRM) mode.

#### Data analysis

To compare the results of the pharmacokinetic study according to administration route, the DPZ concentrations in plasma and brain ISF samples were determined using an LC-MS/MS analysis and plotted over time. The area under the curve in plasma concentration-time curves (AUC_plasma_) and brain ISF concentration-time curves (AUC_ISF_) were determined by non-compartmental analysis using WinNonlin 2.1 (Pharsight, Mountain View, CA). The DPZ concentrations in the whole brain (C_br_), dissected brain, dura and skull were determined.

### Behavior test

#### Classification of animals

Mice were randomly divided into four groups (n = 6-7 per each group): (1) normal control group (NOR), which was administered orally with saline alone; (2) negative control group (NC), which was administered intraperitoneally with scopolamine and orally with saline; (3) positive control group (PC), which was administered intraperitoneally with scopolamine and orally with free DPZ solution (5 mg/kg); (4) test group (DPZ@LAM), which was administered intraperitoneally with scopolamine and intracalvariosseously with DPZ@LAM solution (5 mg/kg as DPZ). Treated drugs (saline for NOR and NC groups, DPZ solution for PC group and DPZ@LAM solution for test group) were once given 1 h before a first novel object recognition test (NORT). 30 min before all the trial, animals also administered saline (i.p., in NOR group) or scopolamine (2 mg/kg, i.p., in NC, PC and DPZ@LAM group).

#### Analgesic protocol

Mice were subcutaneously administered with tramadol hydrochloride (Trodon Injection, Ajupharm, Seoul, Republic of Korea) 20 min before surgery at a dose of 25mg/kg, followed by tramadol in drinking water at a concentration of 0.2 mg/mL continuing for 24 h. From next day after surgical manipulation, the mice received tramadol hydrochloride 25mg/kg at 24h until 14 days by subcutaneous. All mice were housed in an isolated clean cages and provided supplemental heat support until fully ambulatory after skull thinning surgical manipulation.

#### Y-maze

The Y-maze test was performed by modified Yamada's protocol 41. Spatial perception was assessed using by Y-maze test (20 cm long, 10 cm high and 5 cm wide). The three arms of the maze were designated A, B and C. Mice were dropped into the center of the maze and 2 minutes adaptation, observed for 5 minutes to determine the number of times they crossed over each arm. 1 point was given for the mouse entered three different arms consecutively, no score if the entered were not consecutive. After each testing phase, inside the maze was wiped with 70% alcohol to remove excreta and odors. Behavior tests were analyzed by using the SMART v3.0 video tracking system (Panlab, Spain). Spontaneous alternating rate was defined as three consecutive entered on three different arms of the maze. Spatial perception activity was calculated according to the following formula.

Spontaneous Alternation (%) = *[(Number of alternations) / (Total arm entries - 2)] × 100*

#### NORT

The novel object recognition test (NORT) was an experiment that judged memory ability. Test was performed according to the procedures described by Lam *et al.* 42. This test is performed in an open square open box with dimensions of 40 cm × 40 cm × 40 cm and exploration time recorded. First day, the mice were placed in an open square box with two identical objects (A1 and A2) and were free to explore within 5 min. Twenty-four hours later, in the testing phase, one of the two identical objects (A2) was substituted with a novel object (B1) that presented different shape but same texture. Each object was placed about 10 cm away from the wall. The mice were placed in an open box with two identical objects, the mice were free to explore within 5 min. Exploration is defined as the moment the mice approach or touch the object. After each testing phase, all test objects were wiped with 70% alcohol to remove odors and excreta to mice. Behavior tests were analyzed using the SMART v3.0 video tracking system (Panlab, Spain). Novel objective performance was calculated according to the following formula 43, 44.

Preferential index (% time)* = B1/(B1 + A1) × 100*

Discrimination index = *(B1-A1)/(B1 + A1) × 100*

#### Acetylcholinesterase (AchE) activity analysis

Total AchE activities were determined in the mice brain and plasma samples using Ellman's AchE activity method 45. In brief, AchE activity was measured in 0.1M phosphate buffer, pH 7.0, acetylthiocholine iodide solution (75 mM), and buffered Ellman's reagent (10 mM), 5,5-dithio-bis(2-nitrobenzoic acid). The total mixture was incubated for 30 min for reaction at room temperature. Absorbance was measured at 412 nm using a spectrophotometer (Molecular Devices; San Jose, CA, USA). Enzyme activity was expressed as % of normal control group.

#### Histological and immunohistochemical (IHC) analysis

Each mouse collected on the last day of the experiment the mice brain tissues were fixed with 10% neutral-buffered formalin for 24 hour. Brain tissues were embedded in paraffin blocks and sectioned into 6 μm thickness and transferred to slides. Hematoxylin and eosin (H&E) staining and BDNF IHC stains were used to stain the deparaffinized using xylene and dehydrated with EtOH (100%, 90%, 80%, and 70%) brain sections. These sections were incubated with the primary antibody, BDNF (dilution 1 : 500), at 4°C. The stained brain tissue was observed under a Nikon Eclipse 80i Microscope (Tokyo, Japan) at 100× magnification. To quantify the BDNF positive area, we used the ImageJ analyze particles function, which recorded the count and total area of particles. BDNF positive area was normalized as a percentage of total particle area to the total area of the region of interest.

### Complete blood count and biochemistry test

Blood samples collected from the mouse at day 28 after ICO of DPZ@LAM or saline were used for complete blood count (CBC) and biochemistry test. CBC in the blood and the amounts of aspartate aminotransferase (AST), alanine aminotransferase (ALT), γ-glutamyl, alkaline phosphatase (ALP), albumin, globulin, blood urea nitrogen (BUN), and creatinine in the supernatant were analyzed via HLB BioStep (Incheon, Korea).

### Statistical analysis

All data are presented as mean ± SEM. Each value represents the mean of four separate experiments for each group. Significant differences were analyzed using one-way and two-way ANOVA with Turkey's post hoc test. Statistical significance was indicated by *p < 0.05, **p < 0.01 and ***p < 0.001.

## Results

### Preparation and characterization of DPZ@LAM

**Figure 2:**
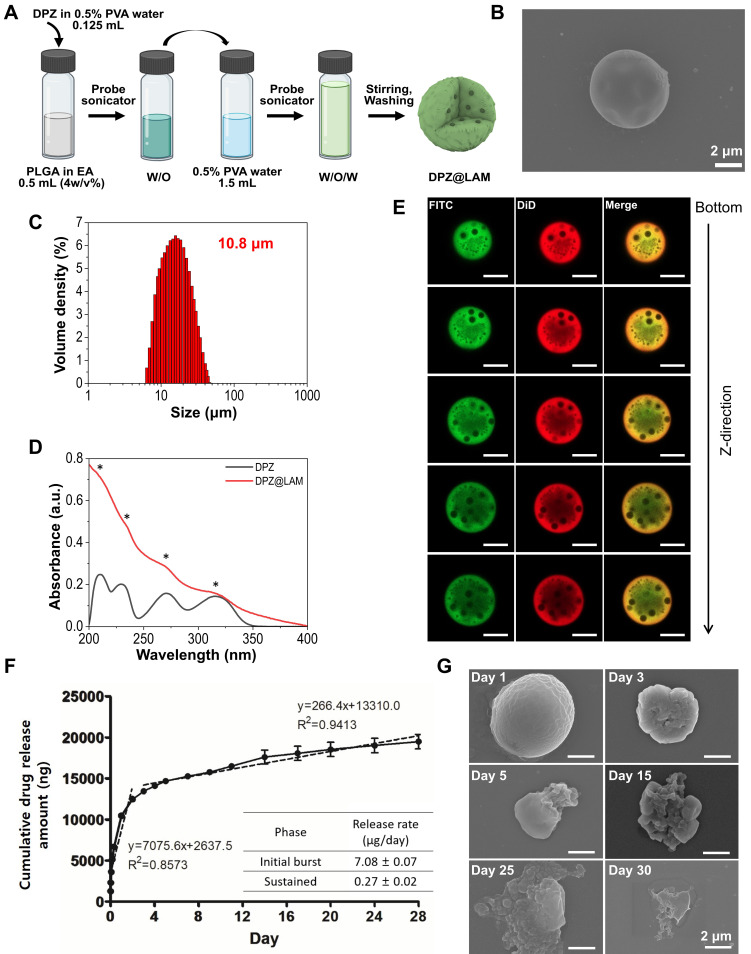
Preparation and characterization of DPZ@LAM. **(A)** Schematic representation of DPZ@LAM by double emulsion-solvent method. **(B)** SEM images of DPZ@LAM. **(C)** Particle size distribution of DPZ@LAM. **(D)** UV/Vis absorbance spectrum of free DPZ and DPZ@LAM showing four characteristic absorption peak (*) of DPZ. **(E)** Confocal microscope z-stack projections of a PLGA microsphere showing the distribution of FITC (log P = 4.5, green) and DiD (log P = ~20, red) dyes. Scale bars = 20 µm. *In vitro* release kinetics and PLGA microspheres erosion analysis. **(F)** Cumulative release of DPZ over 28 days from DPZ@LAM microsphere of 1 mg. Drug release study was performed under sink conditions in 10 mM PBS pH 7.4 at 37°C with 50 rpm (n = 4). **(G)** SEM images of a PLGA microsphere depending on the time of storage in PBS 7.4 solution at 37°C.

DPZ@LAM was synthesized by a water-in-oil-in-water (W_1_/O/W_2_) double emulsion method (Figure 2A). Briefly, first water in oil (W_1_/O) emulsions were prepared via homogenization of biphasic solution consisting PLGA in ethyl acetate mixed with DPZ aqueous solution containing poly(vinyl alcohol) (PVA), as a surface passivating agent. Second emulsion was sonicated in PVA aqueous solution. During the reaction, molecular weight (MW) of PLGA, the concentrations of PVA, and power of homogenization were varied to identify the optimal conditions for the formation of DPZ@LAM with suitable size distribution for ICO. Specifically, the effect of MW of PLGA on microparticle synthesis was investigated (Figure S1A and B). DPZ@LAM prepared with PLGA of 7,000~17,000 MW showed a narrow size distribution and spherical shape, whereas a larger MW of PLGA resulted in irregular shape and broad size distribution of DPZ@LAM. The effect of PVA concentrations was further explored at a pre-determined MW of PLGA (7,000~17,000). As the concentrations of PVA increased from 0.5 to 2%, small sizes of DPZ@LAM were more synthesized, resulting in polydisperse particle mixture (Figure S1A and C). After further optimization, guided by homogenization powers (Figure S1D), the optimal conditions for DPZ@LAM were identified as follows: MW of PLGA (7,000~17,000), PVA concentration (0.5 %), and homogenization power (285 W).

The SEM images of DPZ@LAM prepared with the identified optimal synthetic conditions revealed the smooth and perfectly spherical morphology (Figure 2B and S2). The particle size distribution of DPZ@LAM was narrow and monodispersed with an average size of around 10.8 μm (Figure 2C). DPZ@LAM exhibited characteristic UV/Vis absorption peaks of DPZ, indicating successful loading of DPZ into PLGA microspheres (Figure 2D). The encapsulation efficiency and loading capacity of DPZ in PLGA microspheres were obtained as 36.8 % and 4.0 %, respectively.

Next, location of loaded drug in PLGA microspheres was identified using two different types of fluorescent dye which were chosen based on octanol/water partition coefficient (log P_ow_) value. Since DPZ@LAM was synthesized by W_1_/O/W_2_ double emulsion method, the internal distribution of the drug might be different depending on the hydrophilicity/lipophilicity of the drug (Figure 2E). To provide more detailed investigation of intraparticle drug distribution, we utilized z-stack confocal laser scanning microscopy. PLGA microspheres containing both FITC (λ_ex_ = 488, λ_em_ = 525), which has a similar log P_ow_ value to DPZ (4.5), and lipophilic DiD (log P_ow_ = ~20, λ_ex_ = 648, λ_em_ = 670) were prepared for fluorescence analysis. As shown in Figure 2E, FITC was found to be more present in the center of the microparticles than the lipophilic DiD. FITC was dispersed in water phase during the first W_1_/O emulsion step of the double emulsion process and then, could be located in the inner space of PLGA microspheres. In addition, FITC was distributed relatively evenly within the particles due to its hydrophobic nature. From these results, it can be inferred that DPZ is evenly distributed from the center of the PLGA microspheres, which could result in continuous and long-term release of DPZ until complete degradation of PLGA.

### *In vitro* DPZ release and PLGA microspheres erosion

DPZ@LAM showed two different release kinetics which are biphasic drug release profiles including burst release and sustained phase (Figure 2F). DPZ@LAM reached 100 % release by day 28. The release rate of DPZ in initial burst phase within the zero order kinetics was 7.08 ± 0.07 µg/day over 2 days and in sustained phase with release rate of 0.27 ± 0.02 µg/day for 26 days. It can be expected that DPZ on the surface of the PLGA microspheres, which is not placed in the porous structure of the microsphere, poured out over 2 days and was then released at a constant rate from the PLGA microspheres for 26 days. The data showed that the DPZ@LAM has the capability of a month-long and precisely controlled release performance.

Erosion of the DPZ@LAM was reflected by the SEM micrographs that were collected during the 30 days of the decomposition study as shown in Figure 2G. We observed the morphology of DPZ@LAM at different time points during the storage in 1X PBS 7.4 solution at 37°C. DPZ@LAM was gradually degraded from the surface over time, and after storage for 30 days, it was mostly decomposed, resulting in bulk erosion that altered the shape and surface of the particles. Additionally, it was confirmed by weight measurement after freeze-drying that approximately 50 % of DPZ@LAM stored in PBS remained after 15 days (data not shown). This result indicates that DPZ@LAM could release DPZ for 30 days, which is consistent with the observed result in release test.

### Long-lasting brain exposure of DPZ after a single ICO of DPZ@LAM for four weeks

To assess brain exposure of DPZ, we performed microdialysis study after drug administration with an *in vivo* microdialysis recovery test. The *in vivo* microdialysis recovery rate (R_in vivo_) showed generally stable during 150 min with values 2.50 ± 0.15 % for DPZ. Brain ISF concentration (C_ISF_) was calculated from measured dialysate concentration (C_dialysate_) and R_in vivo_.

**Figure 3:**
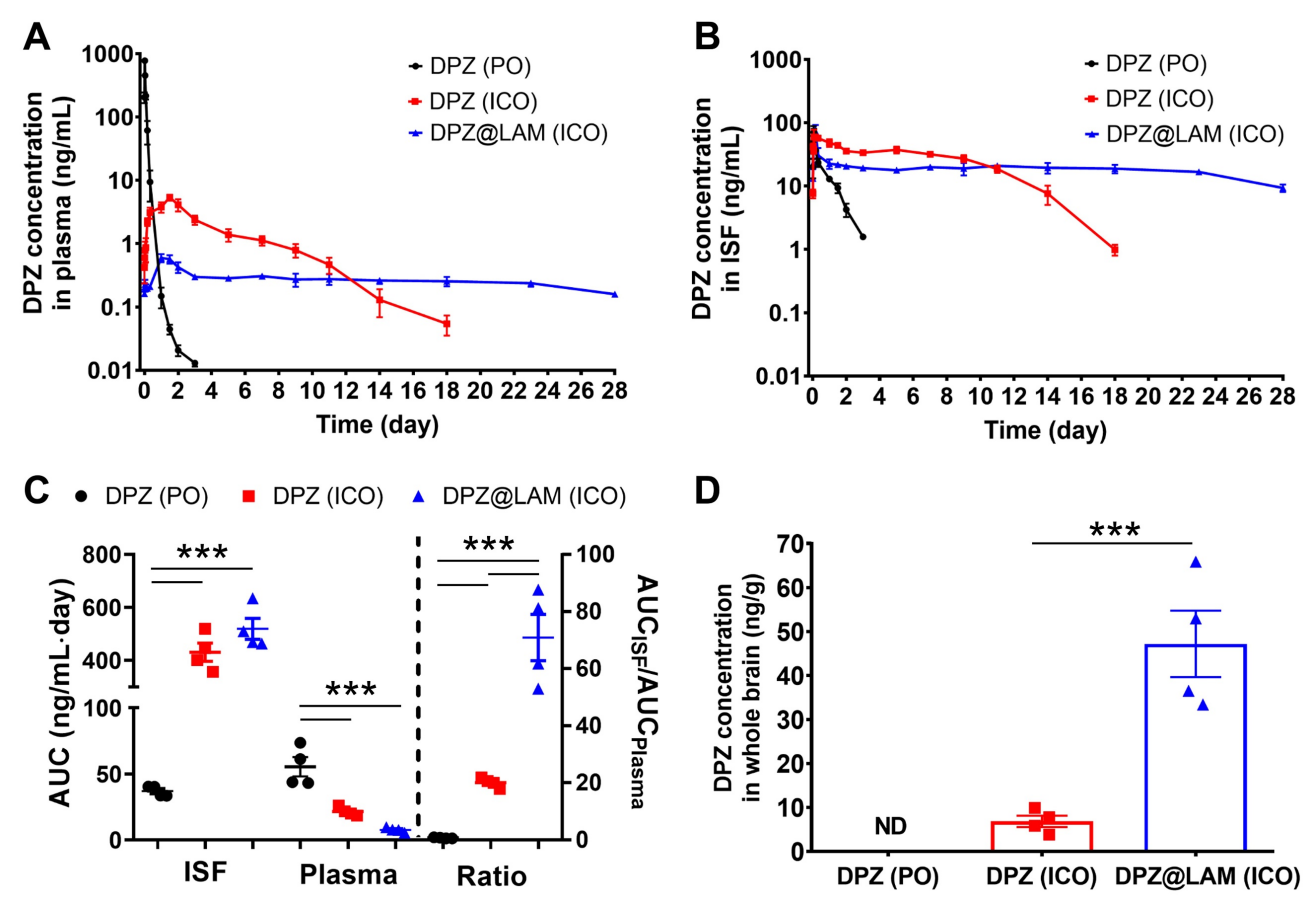
Brain exposure of DPZ after ICO of DPZ@LAM for four weeks in mice as compared to those after ICO and PO of free DPZ. The concentration-time profiles of DPZ in the **(A)** plasma and **(B)** brain ISF. **(C)** Brain to plasma AUC ratio (AUC_ISF_/AUC_plasma_). **(D)** DPZ concentration in whole brain (C_br_) at day 28. Statistical significance was indicated by ***p < 0.001.

To evaluate *in vivo* drug release kinetics and brain exposure of DPZ after ICO of DPZ@LAM, pharmacokinetic studies were carried out following a single ICO of DPZ@LAM, and compared to that of free DPZ (Figure 3). A control group receiving free DPZ via oral administration (PO) group at the same dose was included for comparison. Preliminary estimates for PK parameters were obtained through non-compartmental analysis (NCA) using WinNonlin 2.1 on each PK profile. Table 1 summarizes the pharmacokinetic parameters after ICO of DPZ@LAM and free DPZ with a control group. The control PO group showed a rapid decline in plasma and brain ISF concentrations after 0.5 and 1.5 h, respectively, with DPZ subsequently eliminated within 3 days from both plasma and brain ISF. In ICO of free DPZ group, plasma concentration slowly increased for two days, while brain ISF concentration peaked at 4 h. Subsequently, a decrease was observed in both plasma and brain ISF until day 18. The mean concentration-time profiles of plasma and brain ISF revealed a multiphasic elimination after ICO of DPZ@LAM. The peak concentration of DPZ in plasma was observed at 24 h, whereas that in brain ISF at 4 h. After an initial rapid decline in both plasma and brain ISF concentrations, DPZ exhibited sustained plasma and brain ISF concentrations over 24 days at a constant level and then slowly decreased until day 28. It is shown that the precisely controlled and long-term release capability of DPZ@LAM was also implemented in an* in vivo* condition, consistent with the *in vitro* drug release results.

AUC_plasma_ for four weeks after ICO of free DPZ was 2.6-fold lower than that after PO, whereas AUC_ISF_ for four weeks after ICO of free DPZ was 11.7-fold higher than that after PO (Figure 3C). ICO of DPZ@LAM showed 2.8-fold lower AUC_plasma_ but similar AUC_ISF_ compared to ICO of free DPZ. The highest AUC ratio of 70.87 ± 8.12 was observed in ICO of DPZ@LAM, indicating a 101.2-fold and 3.5-fold increase compared to PO (0.70 ± 0.09) and ICO (20.05 ± 0.84) of free DPZ, respectively. Additionally, the concentration of DPZ in the whole brain (C_br_) after ICO of DPZ@LAM was 47.21 ng/g whereas that of free DPZ was 6.86 ng/g at day 28 post-administration (Figure 3D). Conversely, C_br_ after PO of free DPZ was not detected at day 28.

**Figure 4:**
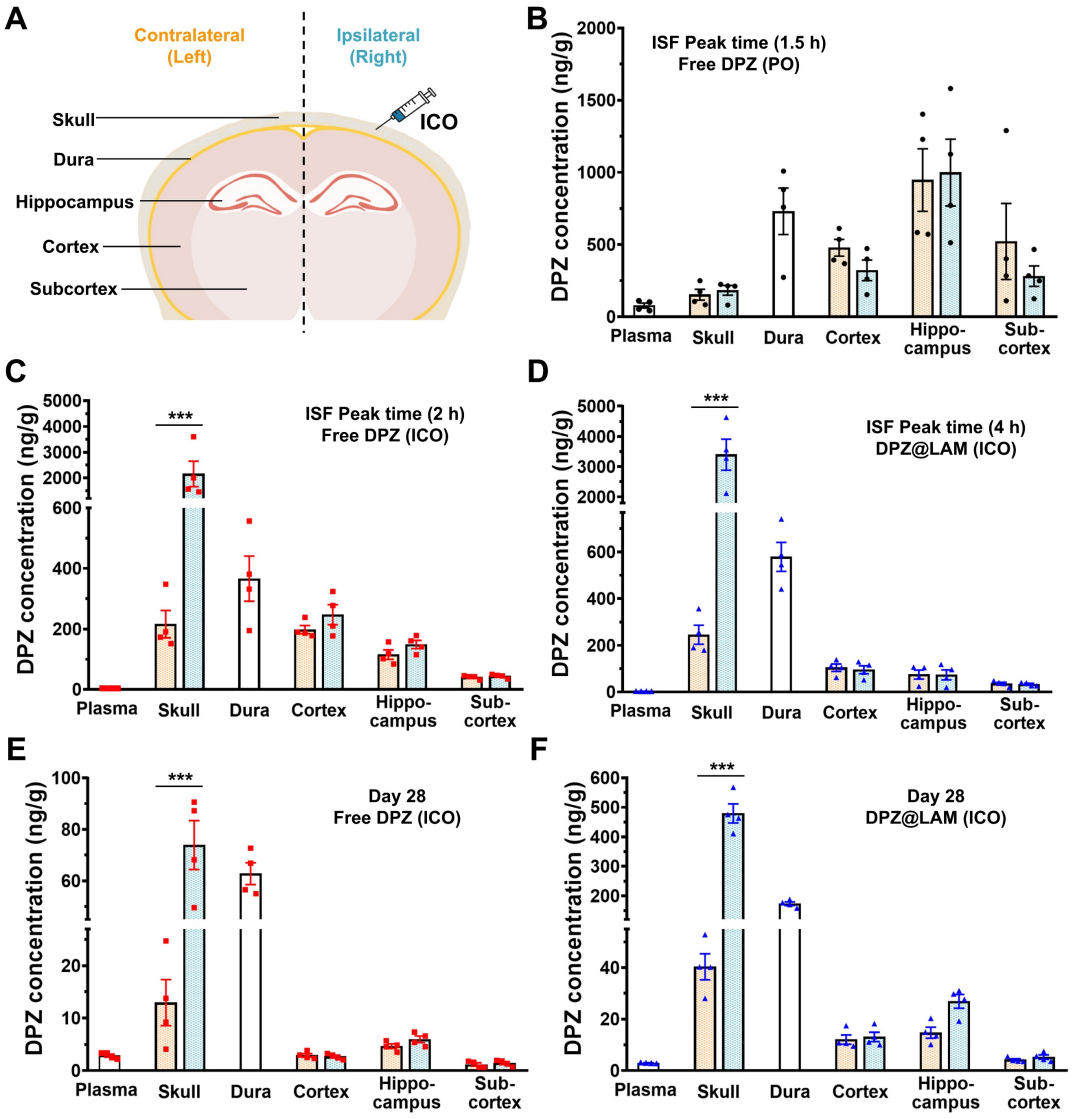
Biodistribution of DPZ in the plasma, skull, dura and brain after treatment. To investigate distribution of DPZ in different brain regions following ICO of free DPZ and DPZ@LAM, the brain distributions were analyzed in dissected cortex, hippocampus and subcortex. **(A)** Schematic demonstrating regions of interest selection, including skull, dura and different brain regions, for assessment of DPZ distribution. Biodistribution of DPZ in the plasma, skull, dura and brain at peak time in the ISF profiles after **(B)** PO of free DPZ, **(C)** ICO of free DPZ and **(D)** ICO of DPZ@LAM. Biodistribution of DPZ in the plasma, skull, dura and brain at day 28 after ICO of **(E)** free DPZ and **(F)** DPZ@LAM. Orange and aqua bars represent the contralateral (left) and ipsilateral (right) sites, respectively. Statistical significance was indicated by ***p < 0.001.

To further study DPZ distribution in the skull, dura, different brain regions after treatment, we carried out biodistribution analysis with dissected skull, dura, cortex, hippocampus and subcortex (Figure 4). Table 2 summarizes the DPZ concentration in each plasma and tissue samples at ISF peak time and day 28 after ICO of DPZ@LAM and free DPZ with a control group (PO). DPZ distribution in relevant tissue and brain regions was analyzed both at day 28 and at the brain ISF peak time to allow for a more accurate comparison. As shown in Figure 4 B, the highest DPZ distribution was observed in the hippocampus with no significant difference between the left and right sides of the skull and each brain regions after a single PO of DPZ at the ISF peak time (1.5 h). However, single ICO of both free DPZ and DPZ@LAM group resulted in the highest DPZ distribution in the cortex at ISF peak time 2 h and 4 h, respectively (Figure 4 C and D). Interestingly, ICO group showed higher DPZ concentrations in the dura compared to the brain. These results suggest that drug distribution in the brain after ICO, which primarily utilizes direct channels between the skull bone marrow and the meninges for brain drug delivery, would be processed differently from that after PO.

DPZ concentrations in the ipsilateral (right) skull, where ICO was applied, were 10-fold and 14-fold higher than those in the contralateral (left) skull in ICO of free DPZ and ICO of DPZ@LAM group, respectively. In contrast, no significant difference was observed between right and left skull in single PO of free DPZ group. Similarly, DPZ concentrations in the right skull were 5-fold and 12-fold higher than those in the left skull at day 28 in ICO of free DPZ and ICO of DPZ@LAM group, respectively (Figure 4E and F). Additionally, ICO of DPZ@LAM group showed 5-fold and 3-fold higher DPZ concentration in the skull and dura, respectively, compared to ICO of free DPZ group. DPZ was not detected in the PO group at day 28 due to complete excretion following a single administration. These results indicated that brain exposure following ICO might be handled differently compared to systemic administration (PO), which relies only on the BBB passing route for brain exposure of DPZ. Furthermore, the sustained release capability of DPZ@LAM provided prolonged and consistent opportunity for brain exposure to DPZ for a month.

### Long-lasting cognitive improvement effect after a single ICO of DPZ@LAM in scopolamine-induced memory deficient mice

**Figure 5:**
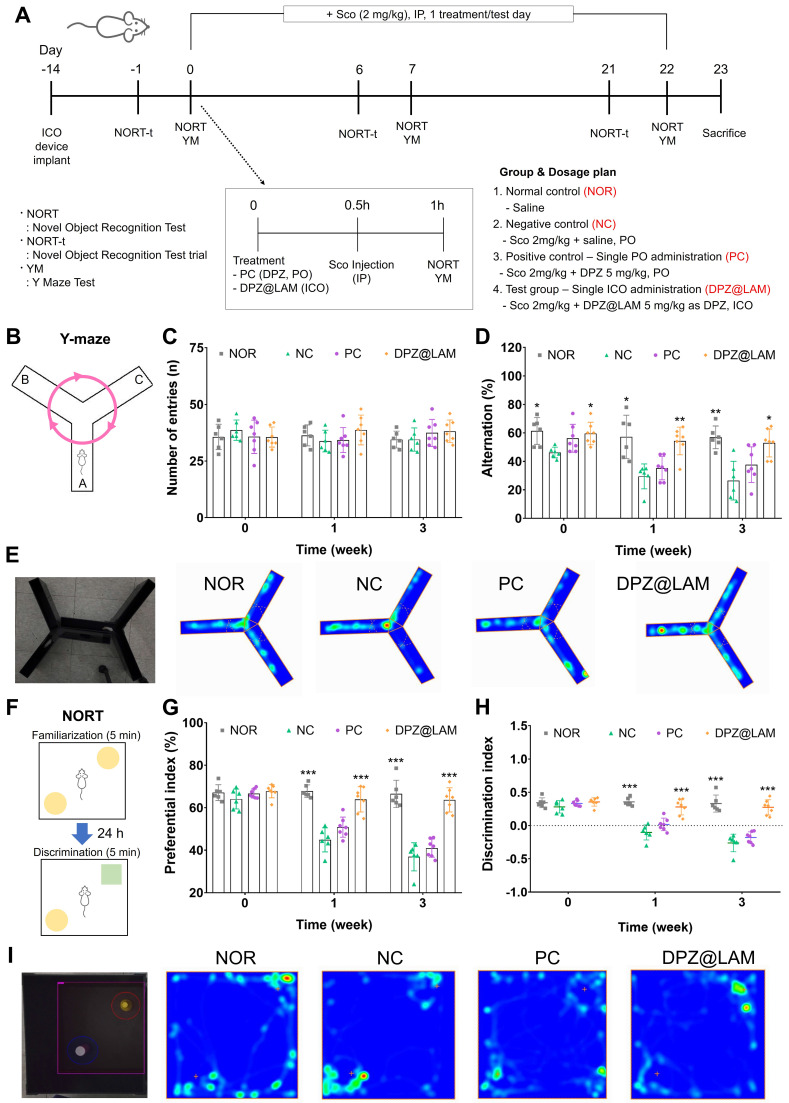
Long-lasting cognitive improvement effect after a single ICO of DPZ@LAM in scopolamine (Sco)-induced memory deficient mice. The mice were intraperitoneally single administered with scopolamine (2 mg/kg) in each test day except normal control (NOR) group. The positive control group (PC) and test group (DPZ@LAM) were treated with DPZ by PO and DPZ@LAM by ICO at a dose of 5 mg/kg as DPZ, respectively. **(A)** Time schedule of the experimental and treatment designs of behavior test. **(B)** Schematic diagram of Y-maze test. **(C)** The number of arm entries on the Y-maze test. **(D)** Alternation of 5 min session on the Y-maze test. **(E)** Representative photograph of the Y-maze test and trajectory heat map images of mouse positions in the experimental arena. **(F)** Schematic diagram of NORT. **(G)** Preferential index and **(H)** discrimination index of 3 min session on the NORT. **(I)** Representative photograph of the NORT and trajectory heat map images of mouse positions in the experimental arena. The data were represented as mean ± SEM (n = 6 - 7 per group) compared with the negative control (NC) group. The differences among the multiple groups were considered by a two-way ANOVA with significant *p < 0.05, **p < 0.01, and ***p < 0.001.

To evaluate the long-acting cognitive improvement effect after a single ICO of DPZ@LAM, behavior tests were carried out by Y-maze and novel object recognition activity assay on scopolamine-induced memory dificient mice (Figure 5). It is well documented that scopolamine, selective muscarinic antagonists, affect the *in vivo* acetylcholine release and memory performance of mice 46, 47. Scopolamine-induced memory dificient mice were prepared through IP of scopolamine (2 mg/kg), followed by an evaluation of its behavioral and biochemical effects on the mice. In accordance with the clinical administration regimen of DPZ, mice were orally administered with a free DPZ solution at an equivalent single dose employed in the pharmacokinetic study of DPZ@LAM (5 mg/kg as DPZ) and assigned as the positive control group.

As shown in Figure 5A, behavior tests, including Y-maze and NORT of three times, were experimental designed and performed after drug administration. Y-maze results are presented in Figure 5B-E. The results of all tests did not reveal significant differences (p>0.05) between the groups in terms of the total number of entries (Figure 5C).The repetitive scopolamine injection caused short-term to long-term memory dysfunction by significantly decreasing the percent of alternation to 26.4 % at week 3 in NC group compared to NOR group of 56.86 ± 3.26 at week 3 (Figure 5D and E, S3). PC group showed the percent of alternation of 56.2 %, 35.2 % and 37.7 % at week 0, 1 and 3, respectively, meaning temporary memory-enhancing at the only treatment day of DPZ and then progression of memory dysfunction by scopolamine injection for 3 weeks. However, a single ICO of DPZ@LAM group showed sustained alternations of 59.6 %, 54.4 % and 52.9 % for 3 weeks, indicating that ICO of DPZ@LAM contributed to long-lasting cognitive improvement effect by prolonged brain exposure.

The results of NORT are presented in Figure 5F-I and S4. Object preferential and discriminal index were calculated by analyzing the number of explorations of familiar and novel objects. Significant difference in the exploration time of familiar and novel objects were observed within the treatment groups. NOR group maintained a preferential index of approximately 67 % and dicrimination index of 0.33 for 3 weeks. Compared to NOR group, NC group showed a significantly decrease in both preferential (37 %) and discrimination (-0.26) index at week 3. PC group initially showed performance similar to the NOR group at week 0 but experienced a decline in preferential and discrimination index at week 1 and 3. Similar to the Y-maze results, this suggests temporary memory enhancement at the only treatment day of DPZ, followed by the progression of memory dysfunction by scopolamine for 3 weeks. On the other hand, ICO of DPZ@LAM group showed sustained maintenance of preferential and discrimination index for 3 weeks. Additionally, ICO of DPZ@LAM group exhibited a preferential index of 64 % and a discrimination index of 0.27 at week 3, sighnificantly increased scales compared to NC group (p<0.001), indicating notable improvements in memory function. Consistent with the finding from the Y-maze test, these results indicate that ICO of DPZ@LAM protects cognitive impairment for a month.

### Alleviation of neuropathology and inhibition of AchE activity after a sinlge ICO of DPZ@LAM in scopolamine-induced memory deficient mice

**Figure 6:**
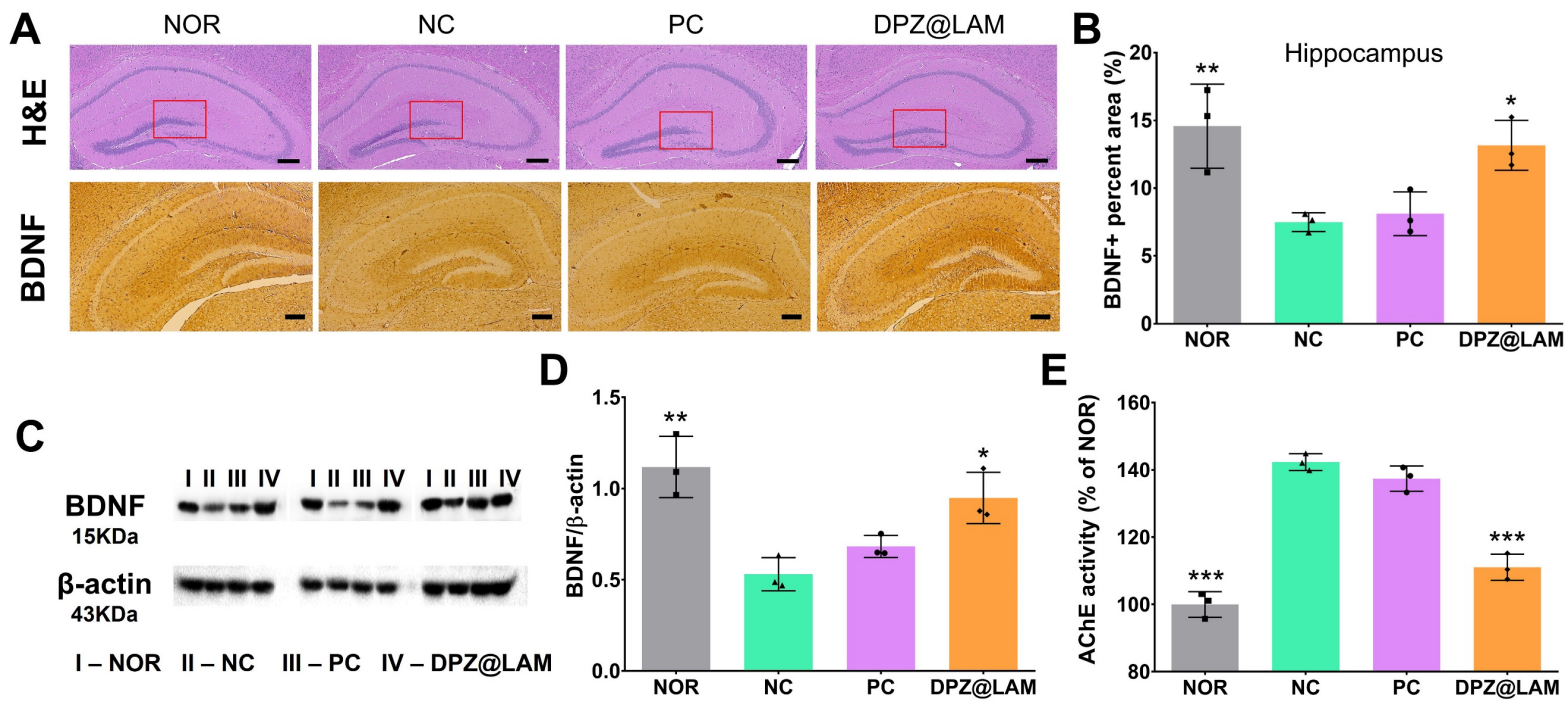
**(A)** Representative photomicrographs of immunohistochemistry in hippocampal tissue of mice in each group after behavior test by hematoxylin and eosin (H&E) and brain-derived neurotrophic factor (BDNF) staining. The scale bars indicate 200 μm. **(B)** The BDNF positive (BDNF+) percent area in the hippocampus of each group. **(C)** Western blot results showing the expression level of BDNF in the brain homogenates of each group on week 3; the β-actin was used as an internal reference. **(D)** Relative levels of BDNF by quantification of western blot results. **(E)** Acetylcholine esterase activity test in brain tissue homogenates after behavior test. (mean ± SEM, n = 3). The data were represented as mean ± SEM (n = 3 per group) compared with the negative control (NC) group. The differences among the multiple groups were considered by a one-way ANOVA with significant *p < 0.05, **p < 0.01, and ***p < 0.001.

Neuronal damage, as evidenced by histopathological examination of hematoxylin and eosin (H&E) stained slides, was observed in the hippocampus, specifically in the dentate gyrus (DG), of sacrificed mice brains at week 3 post administration (Figure 6A and S5). Compared to NC group, it was observed that the number of neurons in NOR group and DPZ@LAM group was significantly higher. It was observed that ICO of DPZ@LAM clear cell borders, increased neuron density, and clear nucleoli in CA3 and CA1, suggesting that ICO of DPZ@LAM could reduce neurodegeneration and recover pathological signs in the hippocampus in scopolamine-induced memory deficient mice.

The number of BDNF stained cells in the CA1 region revealed a significant decrease in the number of stained cells in mice treated with scopolamine, consistent with other studies 48-50 (Figure 6A and S6). BDNF immunoreactivity was notably increased after ICO of DPZ@LAM group compared to that in NC and PC group. It was restored in the CA1 region of the hippocampus of ICO of DPZ@LAM group compared to that in NC and PC group. We then quantified BDNF positive area by BDNF-labeled puncta in the hippocampus (Figure 6B and S7). We found a significant increase of BDNF area in ICO of DPZ@LAM group compared to NC group, resulting in similar levels to those seen in NOR group. Consistent with the findings from the immunohistological imaging analysis, western blot results showed that an increase in BDNF protein levels was observed in ICO of DPZ@LAM group compared to NC group (Figure 6C and D). These result indicate that ICO of DPZ@LAM protects neurodegeneration against scopolamine-induced memory deficient mice for long time duration.

Furthermore, to evaluate the potential mechanisms of ICO of DPZ@LAM in improving cognitive function in scopolamine-induced memory deficient mice, the activities of AchE were measured with sacrificed mice brains at week 3. The increased AchE activity leads to the decrease of acetylcholine, a crucial neurotransmitter involved in cognitive function. As shown Figure 6E, AchE activity was significantly increased in NC group compared to NOR group. ICO of DPZ@LAM group showed markedly reduction in AchE activity, which was increased by scopolamine, compared to NC group. This result suggests that sustained release of DPZ from PLGA microspheres maintains its effectiveness as an AchE inhibitor in the brains of mice.

### *In vivo* biosafety assessment on ICO of DPZ@LAM

To evaluate the *in vivo* biosafety following ICO of DPZ@LAM at administered dose (5mg/kg), we performed serum biochemical and hematological tests in mice. Biochemical and hematological parameters were measured in the blood samples collected on day 28 after ICO of DPZ@LAM or saline to investigate any potential adverse effects specific to the ICO of DPZ@LAM. The control group consisted of four healthy mice that did not receive any treatment. As shown in **Table S1,** we examined various blood biochemical parameters, including AST, ALT, ALP, albumin, globulin, BUN, and creatinine. The results showed that no significant differences in the ICO of DPZ@LAM and saline groups compared to the control group. Similarly, all hematological parameters in both ICO of DPZ@LAM and saline showed no substantial differences from the control group (**Table S2**). These *in vivo* results suggest that DPZ@LAM has biocompatibility at administered dose and that ICO does not cause any toxicity or influence on immune systems of mice over the 4-week periods.

## Discussion

In this study, we report that intracalvariosseous administration (ICO) of donepezil-loaded long-acting microspheres allows prolonged brain exposure in mice, thereby ameliorating cognitive impairment and neurodegeneration. DPZ@LAM, successfully prepared by double emulsion method, showed smooth and perfectly spherical morphology with an average size of 10.8 μm. DPZ@LAM possesses the capability of a month-long and precisely controlled release performance with a release rate of 0.27 ± 0.02 µg/day. A month-long sustained *in vivo* brain exposure after ICO of DPZ@LAM led to long-acting cognitive improvement effect.

Skull bone consists of three-layered structure with a trabecular layer (diploe), sandwiched between two layers of dense cortical bone (cortex). The trabecular separation, indicating the distance among trabecular skeleton structures in the diploe of mice skulls, is approximately 150 µm 51. In case humans, the bone marrow cavities within the diploe are characterized by a minimum length of approximately 0.07 - 0.12 mm and a maximum length of approximately 3.6 - 6.47 mm 52. Considering dimension of bone marrow cavities, DPZ@LAM were prepared with an average size of 10.8 μm (ranging from 6 to 50 μm). The size is sufficiently small to pass through a needle of ~ 33 gauge for injection into the diploic area of the skull.

It was reported that medication compliance is defined as "the extent to which a patient acts in accordance with the prescribed interval and dose of a dosing regimen" 53. Medication compliance is a critical factor for the treatment of chronic neurodegenerative AD and major obstacle in achieving and maintaining therapeutic outcomes 17, given that the medication must be taken continuously for the duration of the patient's life 54. DPZ has long been primarily considered as an agent for symptomatic improvement of AD and is commonly marketed in once-daily oral dosage forms of 5 - 23 mg/tablet. However, the memory impairment in AD patients often results in poor compliance even to the daily dosing regimen 55. Therefore, long-acting dosage forms of DPZ could be a strong strategy for overcoming the poor compliance and enhancing therapeutic outcomes.

Long-acting dosage forms with controlled release capability are currently being developed in various forms such as transdermal patches (Exelon®, Neupro®, Aricept patch®) 56, 57, injectable microspheres (Risperdal Consta®, Bydureon™, Lupron Depot®) 21 and implants (ALZET® osmotic pump, Implanon®, Probuphine®) 58, 59 to address numerous challenges associated with conventional methods of administration 19. Among various implants, implantable pumps provide long-term drug release with well-controlled rate, but need complex fabrication of pump systems and invasive surgical procedure for implantation and removal 19, 60. In our previous study, commercial osmotic pumps were employed and adapted to the newly established ICO route, proposing a new long-acting dosing regimen for various drugs, in particular BBB-impermeable CNS drugs with small to colloidal sizes. In this study, the approach of ICO for long-acting brain drug delivery was further explored to long-acting injectable microsphere, as non-invasive alternative to the pump implant of surgical procedure. We prepared donepezil-loaded long-acting injectable microspheres with biodegradable PLGA (DPZ@LAM), as an alternative to the pump of no surgical procedure. DPZ@LAM was capable of sustained and controlled release with a month-long duration at nano flow rate and then allowed long-lasting brain exposure and cognitive improvement in mice, proving a promising strategy for overcoming the poor compliance and enhancing therapeutic outcomes.

The adverse effects of DPZ by daily oral administration, which are associated with hypercholinergic activity in the central nervous and peripheral organs 11, 17, are primarily influenced by large and frequent fluctuations in plasma drug concentrations 61. To avoid the adverse effects and maintain therapeutic efficacy of DPZ, ICO could be a strong strategy since it allows DPZ to directly reach parenchymal brain with less entering systemic circulation and thus can produce small fluctuations in plasma concentration. A single ICO of free DPZ showed lower peak in plasma concentration but similar peak in brain ISF concentration compared to PO (Figure 3). DPZ after PO should be first absorbed into the blood circulation and then a part of DPZ in the blood enters the brain. However, DPZ after ICO can directly enter the brain and achieve enhanced brain exposure, representing a potent way capable of addressing the limitations inherent in the systemic route. A single ICO of DPZ@LAM showed sustained concentration in brain ISF above the effective therapeutic levels for a month, but PO of free DPZ led to subtherapeutic brain ISF concentration after 4 h. In contrast, plasma concentration after a single ICO of DPZ@LAM was significantly lower compared to that after PO of free DPZ. It can be safely estimated that a single ICO of DPZ@LAM can achieve a therapeutic effect comparable to multiple daily PO dose of free DPZ for 28 days. Altogether, a single ICO of DPZ@LAM can increase brain exposure and reduce the required dose, minimizing adverse effects and thus improving patient compliance in AD treatment.

ICO could be applied to anti-amyloid monoclonal antibodies recently FDA-approved for the treatment of AD such as Aducanumab and Lecanemab. Antibodies, due to their large molecular size, exhibit limited passage across the BBB. Several studies reported brain antibody concentrations of less than 0.1% of the injected dose, being estimated that less than 1 in 1000 antibody molecules successfully reach the brain 62, 63. The limited brain availability of antibodies necessitates administration of high dose to achieve therapeutic concentration of antibodies in the brain. However, the high dosage regimen often results in severe adverse effects. Amyloid-related imaging abnormalities (ARIAs), which are side effects associated with the removal of Aβ, were found to be dose-dependent in the aducanumab-treated group 64. To enhance the efficient passage of monoclonal antibodies across the BBB and maintain sustained levels in the brain, alternative administration routes such as intracerebroventricular (ICV) and intranasal (IN) are being explored 64. ICV provides opportunity to achieve high brain parenchymal drug concentrations but is invasive and are associated with risk of complications 65. IN offers a non-invasive method of bypassing the BBB to deliver drugs to the CNS. However, many challenges associated with IN still remain as follows: i) rapid elimination of drug substances from nasal cavity due to mucocilliary clearance, ii) mechanical loss of the dosage form and low reproducibility resulting from improper administration technique, iii) mucosal damage with frequent use 66, 67. Hence, ICO employs a BBB-independent route for drug entry to the brain and is less invasive process than ICV and IT, may represent a promising strategy to overcome the limitations associated with monoclonal antibody therapeutics. Furthermore, ICO holds promise for the brain drug delivery of potential but BBB-impermeable biologics including nucleic acids, peptides, proteins, viral vectors and even stem cells.

Although ICO serves as a promising approach for brain drug delivery, several potential risks such as infection, hemorrhage and bone damage must be carefully considered to ensure the success of intracalvariosseous-brain drug delivery. Introducing an infection into the bone marrow space can lead to bone marrow inflammation or osteomyelitis 68, 69. Additionally, bleeding into surrounding tissues (hematoma) may occur if the procedure damages blood vessels in the skull 70-72. Improper needle insertion can also cause bone damage, potentially resulting in fractures or other bone-related complications 69, 73. These risks would be primarily associated with surgical processes involved in ICO, such as the skull thinning and device mounting. Numerous studies on ICV administration have demonstrated that employing strict aseptic techniques and adhering to rigorous standard practices can significantly reduce complication rates 74-76. Similarly, the potential risks of ICO can be minimized by implementing proper procedures, including: i) meticulous attention to maintaining aseptic conditions, ii) standardized surgical protocols based on a thorough understanding of skull anatomy, iii) well-trained surgical techniques with careful attention to detail during surgery and the post-operative period, iv) the use of intraoperative or post-operative imaging to confirm placement of needle and detect any hemorrhage, and v) application of an optimized ICO device, considering specifications of the needle inserted into the skull such as materials, gauges, lengths and types 65, 71, 73-80. These procedures could help reduce patient discomfort accompanied intra- and post-operation for ICO, such as extended anesthesia, prolonged pain and the management of complications. Although several limitations remain to be addressed prior to clinical application, we believe that ICO has the potential to open a new chapter for methods for brain drug delivery that circumvent the BBB.

## Conclusion

In this study, we demonstrated that intracalvariosseous administration of donepezil-loaded microspheres protects against cognitive impairment and neurodegeneration by providing sustained brain exposure in mice. DPZ@LAM was successfully prepared via double emulsion method, resulting in microspheres with a smooth and perfectly spherical morphology. DPZ@LAM exhibited precisely controlled release performance lasting one month, both *in vitro* and *in vivo*. Prolonged *in vivo* brain exposure for one month after ICO of DPZ@LAM led to significant cognitive improvement in scopolamine-induced memory-deficient mice, accompanied by inhibited acetylcholinesterase activity and increased brain-derived neurotrophic factor. These findings suggest that ICO allows for BBB-bypassing brain drug delivery through the direct connection between the skull bone marrow and brain, offering an alternative approach for the treatment of neurodegenerative diseases with CNS drugs that are otherwise impermeable to the BBB.

## Supplementary Material

Supplementary figures and tables.

## Figures and Tables

**Table 1:** Pharmacokinetic parameter after ICO of DPZ@LAM for four weeks (mean ± SEM, n = 4).

	Administration route	AUC_plasma_ (ng/mL∙day)	AUC_ISF_ (ng/mL∙day)	AUC ratio (AUC_ISF_/AUC_plasma_)	C_br_ (ng/g)
Free DPZ	PO	55.44 ± 7.37	36.93 ± 1.97	0.70 ± 0.09	ND
	ICO	21.51 ± 1.63	430.75 ± 34.71	20.05 ± 0.84	6.86 ± 1.30
DPZ@LAM	ICO	7.57 ± 0.88	519.00 ± 39.77	70.87 ± 8.12	47.21 ± 7.57

**Table 2:** DPZ concentration in the plasma, skull, dura and brain at day 28 and peak time in the ISF profiles after PO of free DPZ, ICO of free DPZ and ICO of DPZ@LAM (mean ± SEM, n = 4). L and R indicate contralateral (left) and ipsilateral (right), respectively.

Time		Peak time				Day 28		
		1.5 h	2 h	4 h				
		DPZ (PO)	DPZ (ICO)		DPZ@LAM (ICO)	DPZ (PO)	DPZ (ICO)	DPZ@LAM (ICO)
Plasma (ng/mL)		77.96 ± 16.84	3.94 ± 0.16		3.67 ± 0.08	ND	2.86 ± 0.29	2.98 ± 0.09
Skull (ng/g)	L	153.50 ± 37.53	215.90 ± 44.76		245.58 ± 40.74	ND	12.95 ± 4.40	40.31 ± 5.06
	R	183.31 ± 33.93	2156.51 ± 496.08		3399.14 ± 516.27	ND	73.90 ± 9.48	479.11 ± 32.54
Dura (ng/g)		730.54 ± 160.47	366.03 ± 74.65		579.00 ± 62.38	ND	62.79 ± 4.22	173.22 ± 6.17
Cortex (ng/g)	L	478.26 ± 58.13	198.15 ± 13.59		104.16 ± 15.73	ND	2.91 ± 0.34	12.09 ± 1.79
	R	322.32 ± 71.24	247.08 ± 32.96		95.23 ± 17.21	ND	2.69 ± 0.27	13.10 ± 1.81
Hippocampus (ng/g)	L	947.14 ± 215.85	115.29 ± 15.64		74.73 ± 19.44	ND	4.66 ± 0.45	14.71 ± 2.10
	R	998.18 ± 231.43	148.79 ± 13.71		73.52 ± 21.58	ND	5.97 ± 0.62	26.89 ± 2.67
Subcortex (ng/g)	L	521.59 ± 262.91	40.33 ± 3.07		35.85 ± 6.09	ND	1.17 ± 0.30	4.18 ± 0.44
	R	281.48 ± 70.65	44.06 ± 3.21		33.47 ± 3.80	ND	1.44 ± 0.29	5.27 ± 0.85
								
