# Supplementary material for: Intracalvariosseous administration of donepezil microspheres protects against cognitive impairment by virtue of long-lasting brain exposure in mice
Source: Theranostics. 2024 Oct 14;14(17):6708–25. doi: 10.7150/thno.100986 (PMC11519799; doi:10.7150/thno.100986)
Supplement: Supplementary file 1 — Supplementary figures and tables. [file thnov14p6708s1.pdf]

Supporting Information

Intracalvariosseous administration of donepezil microspheres protects against cognitive impairment by virtue of long-lasting brain exposure in mice

Ji Hee Kang[1], Jin-Kyoung Yang[2], Kyo Hee Cho[1], O Hyun Lee[1], Hayoon Kwon[3], Sun Yeou Kim[1], Sehoon Kim[3,4], Young Tag Ko[1*]

[1] College of Pharmacy and Gachon Institute of Pharmaceutical Sciences, Gachon University, Incheon, 21936, Republic of Korea

[2] Department of Chemical Engineering, Dong-Eui University, Busan, 47340, Republic of Korea

[3] Chemical & Biological Integrative Research Center, Korea Institute of Science and Technology, Seoul, 02792, Republic of Korea.

[4] KU-KIST Graduate School of Converging Science and Technology, Korea University, Seoul, 02841, Republic of Korea

[*] Address for correspondence: Professor Young Tag Ko, College of Pharmacy, Gachon University, 191 Hambakmoe-ro, Yeonsu-gu, Incheon, Republic of Korea 21936

Tel.: 82-32-820-4923; Fax: 82-32-820-4829

E-mail address: youngtakko@gachon.ac.kr

Supplemental Results

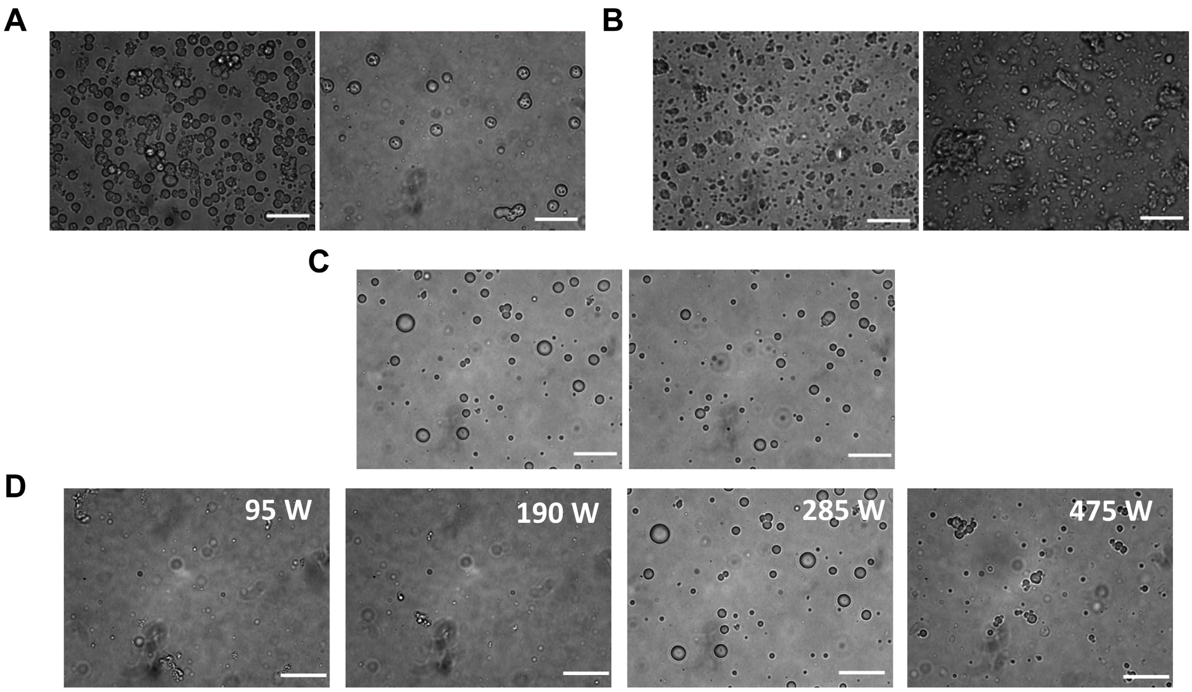

Figure S1. Optimization of DPZ@LAM preparation. The PLGA molecular weight (Mw), PVA concentration, and homogenization power effect on microsphere size and morphology. DPZ@NAM prepared with **(A)** PLGA of Mw 7,000~17,000 in 0.5 % PVA solution, **(B)** PLGA of Mw 24,000~38,000 in 0.5% PVA solution, and **(C)** PLGA of Mw 7,000~17,000 in 2 % PVA solution. **(D)** Effect of homogenization power on microsphere size and morphology.

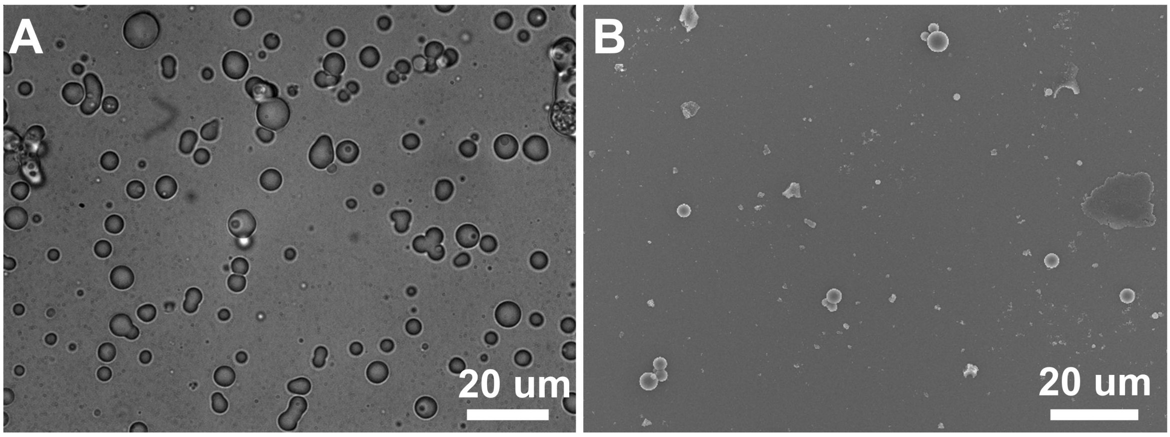

Figure S2. Morphology and size of DPZ@NAM prepared by double emulsion technique. **(A)** Optical microscope image and **(B)** scanning electron microscope images of DPZ@NAM.

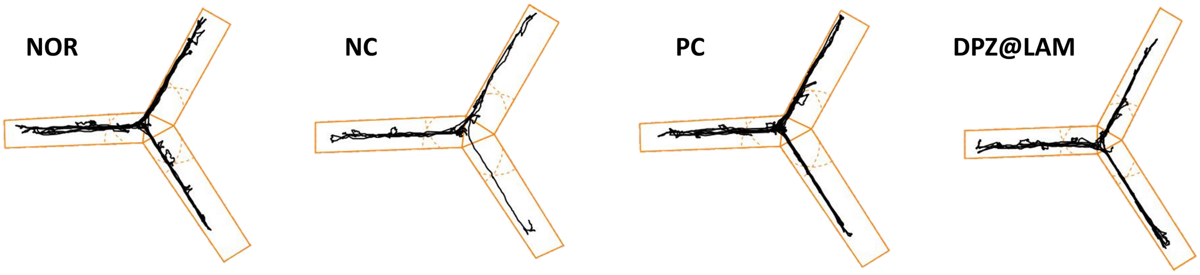

Figure S3. Trajectory charts of each group in the Y-maze test at week 3.

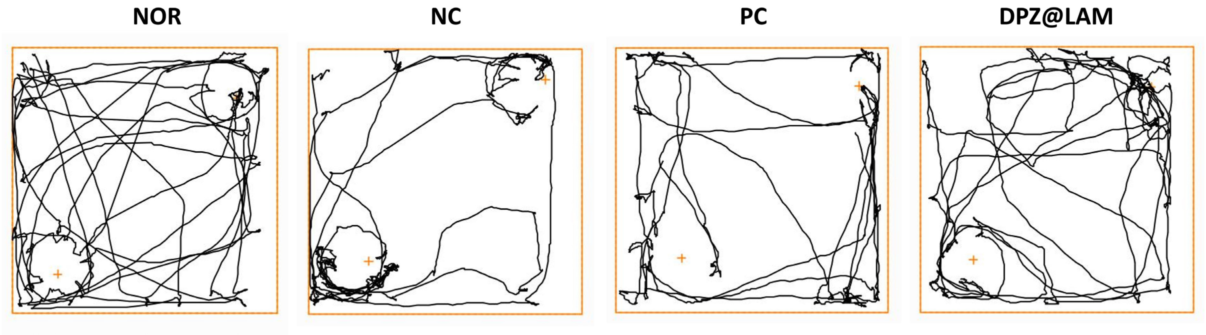

Figure S4. Trajectory charts of each group in the NORT at week 3.

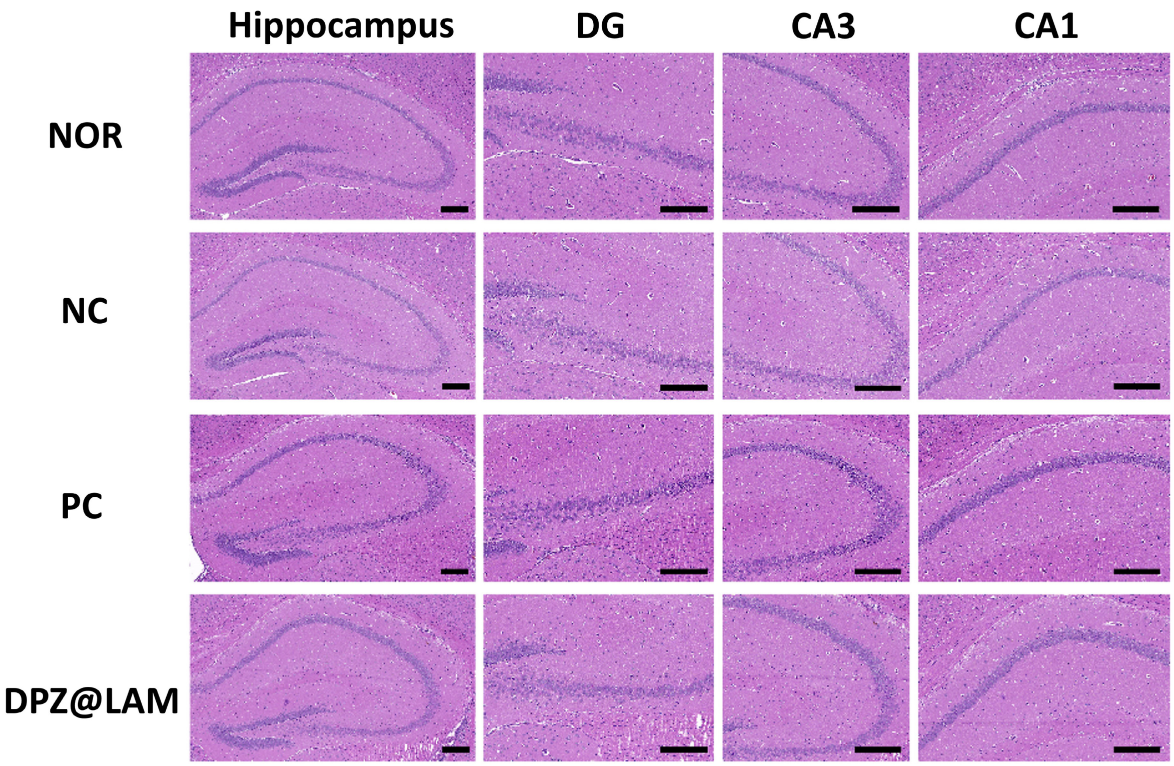

Figure S5. Representative photomicrographs of immunohistochemistry in hippocampal tissue of mice in each group after behavior test. The morphological changes of DG, CA3, CA1 region from hippocampus by hematoxylin and eosin (H&E) analysis. The scale bars indicate 200 μm.

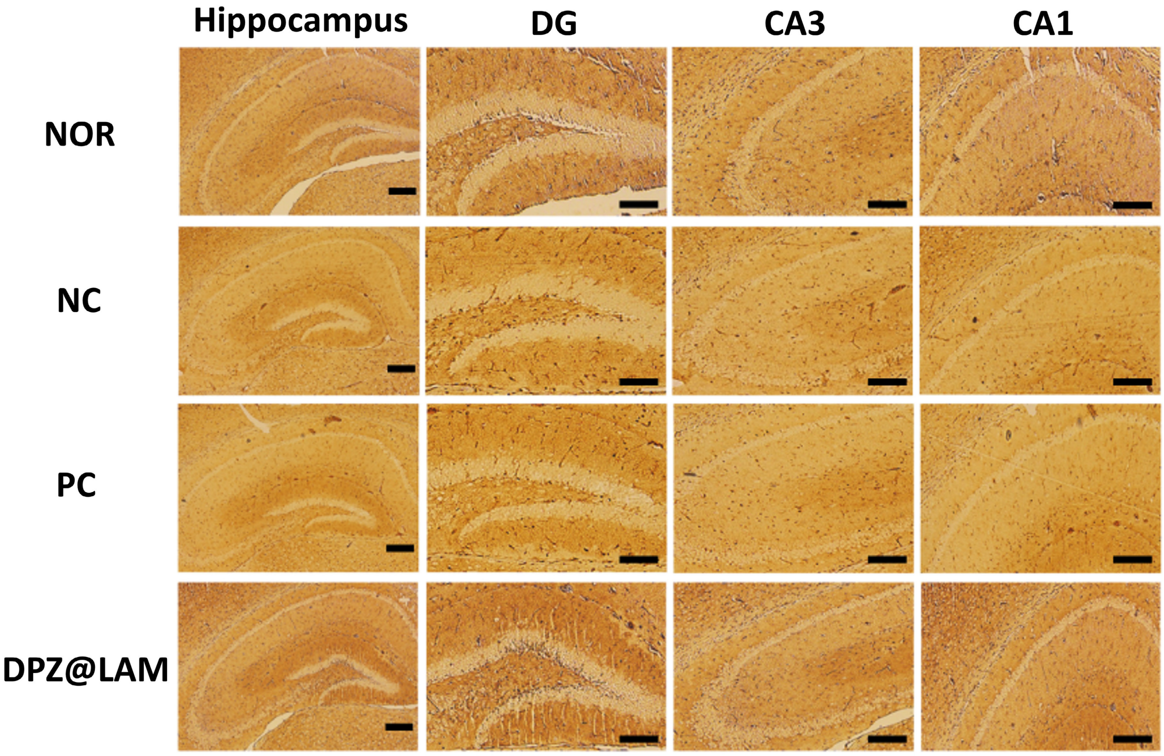

Figure S6. Representative photomicrographs of brain-derived neurotrophic factor (BDNF) expression in hippocampal tissue of mice in each group. The scale bars indicate 200 μm.

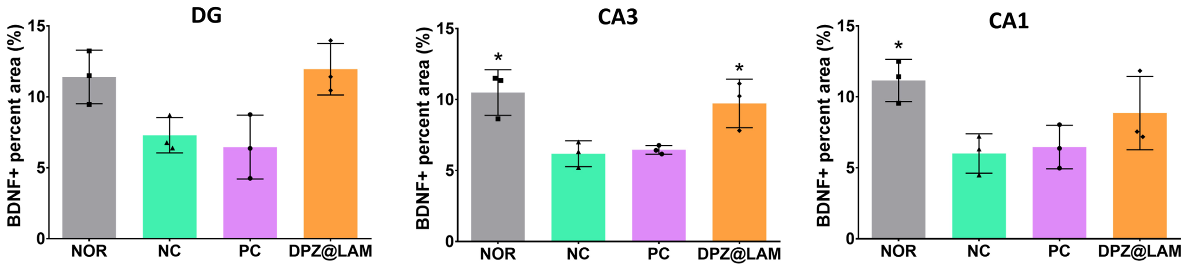

Figure S7. Quantitative analysis of BDNF-positive area in DG, CA3, CA1 region from IHC staining images of BDNF. Data are expressed as means Mean ± SEM, n = 3 and statistical analysis by one-way ANOVA followed by Dunnett test *$p < 0.05$.

Table S1. Biochemical parameters measured in the blood of mice after ICO of DPZ@LAM or saline for 4 weeks (n = 4, mean ± SEM). The results of the control group were obtained from the blood of four healthy mice without any treatment.

Variable (units) [a]	Groups			
	Control	Saline (ICO)	DPZ@LAM (ICO)	Normal Range [b]
AST (U/L)	182.0 ± 29.75	155.3 ± 21.97	143.6 ± 16.46	55 - 352
ALT(U/L)	62.2 ± 7.01	53.9 ± 10.14	54.2 ± 10.71	41 – 131
ALP (U/L)	409.4 ± 9.13	385.9 ± 38.32	414.7 ± 18.72	118 – 433
Albumin (g/dL)	3.28 ± 0.15	2.99 ± 0.40	2.94 ± 0.57	2.7 – 4.6
Globulin (g/dL)	3.12 ± 0.14	3.12 ± 0.09	3.09 ± 0.08	2.7 – 3.5
BUN (mg/dL)	21.51 ± 2.29	23.09 ± 1.27	22.71 ± 0.88	7 – 26
Creatinine (mg/dL)	0.38 ± 0.02	0.39 ± 0.02	0.40 ± 0.02	0.2 – 0.4

[a] aspartate aminotransferase (AST); alanine aminotransferase (ALT); alkaline phosphatase (ALP); blood urea nitrogen (BUN)

[b] Reference intervals for biochemical parameters of Balb/c mouse from literature [1-2].

Table S2. Hematological parameters in the rabbits after ICO of DPZ@LAM or saline for 4 weeks (n = 4, mean ± SEM). The results of the control group were obtained from the blood of four healthy mice without any treatment.

Variable (units) [a]	Groups			
	Control	Saline	DPZ@LAM	Normal Range [b]
WBC (x10^3cells/μℓ)	5.45 ± 0.39	4.13 ± 0.67	4.39 ± 0.51	3.48 – 14.03
RBC (x10^6cells/μℓ)	9.10 ± 0.16	9.69 ± 0.16	9.29 ± 0.62	6.93 – 12.24
HGB (g/dL)	15.3 ± 0.32	15.1 ± 0.27	14.2 ± 0.87	12.6 – 20.5
HCT (%)	46.7 ± 1.71	47.5 ± 1.34	45.0 ± 3.00	42.1 – 68.3
MCV (fL)	48.3 ± 0.79	49.2 ± 0.70	48.5 ± 0.38	50.7 – 64.4
MCH (pg)	15.5 ± 0.14	15.5 ± 0.06	15.3 ± 0.17	13.2 – 17.6
MCHC (g/dL)	31.8 ± 0.09	31.3 ± 0.40	31.6 ± 0.23	23.3 – 32.7
RDW (%)	13.5 ± 0.25	13.6 ± 0.25	14.0 ± 0.26	16.9 – 23.4
PLT (x10^3 cells/μℓ)	798.0 ± 77.90	685.5 ± 105.12	653.5 ± 105.95	420 – 1698
MPV(fL)	10.1 ± 0.51	9.1 ± 0.93	9.6 ± 1.09	4.6 – 5.9
Neutrophils (%)	17.1 ± 2.34	24.5 ± 2.37	28.9 ± 3.74	9.86 – 39.11
Lymphocytes (%)	66.9 ± 3.05	60.8 ± 3.22	61.5 ± 3.79	48.81 – 83.19
Monocytes (%)	1.5 ± 0.18	1.7 ± 0.21	2.1 ± 0.21	0.11 – 4.91
Eosinophils (%)	3.5 ± 0.25	3.4 ± 0.41	3.9 ± 0.35	3.29 – 12.48
Basophils (%)	0.2 ± 0.04	0.3 ± 0.08	0.4 ± 0.11	0.00 – 1.84

[a] white blood cell count (WBC); red blood cell count (RBC); hemoglobin (HGB); hematocrit (HCT); mean corpuscular volume (MCV); mean corpuscular hemoglobin (MCH); mean corpuscular hemoglobin concentration (MCHC); red cell diameter width (RDW); platelets (PLT); mean platelet volume (MPV)

[b] Reference intervals for hematological parameters of Balb/c mice from literature [2].

Supporting References

1. Silva-Santana G, Bax JC, Fernandes DCS, Bacellar DTL, Hooper C, Dias A, et al. Clinical hematological and biochemical parameters in Swiss, BALB/c, C57BL/6 and B6D2F1 Mus musculus. Animal models and experimental medicine. 2020; 3: 304-15.

2. Balb-C mouse data sheet, http://www.criver.com/products-services/ basic-research/find-a-model/balb-c-mouse, accessed: March 2012.
